# Supplementary material for: Emissions mitigation opportunities for savanna countries from early dry season fire management
Source: Nat Commun. 2018 Jun 8;9:2247. doi: 10.1038/s41467-018-04687-7 (PMC5993717; doi:10.1038/s41467-018-04687-7)
Supplement: Supplementary file 2 — Description of Additional Supplementary Information [file 41467_2018_4687_MOESM2_ESM.docx]

**Description of Additional Supplementary Files**

File Name: Supplementary Data 1

Description:

Total mean and standard deviation (SD) of EDS and LDS savanna emissions (tCO2e yr-1) per country. Also shown are the mean (SD) difference between LDS and EDS used to calculate abatement potential in this study. In addition, the number of GFED pixels that intersected savanna habitat in each country are shown. These results are based just on the portion of savanna habitat that received greater than 600 mm of precipitation. For countries that straddle the equator, data are shown for both the portion north and south of equator.

File Name: Supplementary Data 2

Description:

Total areal extent of savanna habitat (km2) and extent of savanna with a mean of >600 mm of precipitation for each country. For countries that straddle the equator, data are shown for both the portion north and south of equator.

File Name: Supplementary Data 3

Description:

The areal extent of protected areas within total savanna habitat and just the portion that receives greater than 600 mm of precipitation for each country. Also shown is the percent of savanna habitat that is designated a protected area. For countries that straddle the equator, data are shown for both the portion north and south of equator.
